# FGF21 augments autophagy in random-pattern skin flaps via AMPK signaling pathways and improves tissue survival

**DOI:** 10.1038/s41419-019-2105-0

**Published:** 2019-11-18

**Authors:** Kailiang Zhou, Huanwen Chen, Jinti Lin, Hui Xu, Hongqiang Wu, Guodong Bao, Jiafeng Li, Xiangyang Deng, Xiaolong Shui, Weiyang Gao, Jian Ding, Jian Xiao, Huazi Xu

**Affiliations:** 1 Department of Orthopaedics, The Second Affiliated Hospital and Yuying Children’s Hospital of Wenzhou Medical University, Wenzhou, 325027 China; 2 Zhejiang Provincial Key Laboratory of Orthopaedics, Wenzhou, 325027 China; 3 University of Maryland School of Medicine, Baltimore, MD 21201 USA; 4 Department of Neurosurgery, The Second Affiliated Hospital and Yuying Children’s Hospital of Wenzhou Medical University, Wenzhou, 325027 China; 5 Molecular Pharmacology Research Center, School of Pharmaceutical Science, Wenzhou Medical University, Wenzhou, 325027 China

**Keywords:** Pharmacology, RNAi, Trauma, Drug development

## Abstract

Random-pattern skin flap is commonly used for surgical tissue reconstruction due to its ease and lack of axial vascular limitation. However, ischemic necrosis is a common complication, especially in distal parts of skin flaps. Previous studies have shown that FGF21 can promote angiogenesis and protect against ischemic cardiovascular disease, but little is known about the effect of FGF21 on flap survival. In this study, using a rat model of random skin flaps, we found that the expression of FGF21 is significantly increased after establishment skin flaps, suggesting that FGF21 may exert a pivotal effect on flap survival. We conducted experiments to elucidate the role of FGF21 in this model. Our results showed that FGF21 directly increased the survival area of skin flaps, blood flow intensity, and mean blood vessel density through enhancing angiogenesis, inhibiting apoptosis, and reducing oxidative stress. Our studies also revealed that FGF21 administration leads to an upregulation of autophagy, and the beneficial effects of FGF21 were reversed by 3-methyladenine (3MA), which is a well-known inhibitor of autophagy, suggesting that autophagy plays a central role in FGF21’s therapeutic benefit on skin flap survival. In our mechanistic investigation, we found that FGF21-induced autophagy enhancement is mediated by the dephosphorylation and nuclear translocation of TFEB; this effect was due to activation of AMPK-FoxO3a-SPK2-CARM1 and AMPK-mTOR signaling pathways. Together, our data provides novel evidence that FGF21 is a potent modulator of autophagy capable of significantly increasing random skin flap viability, and thus may serve as a promising therapy for clinical use.

## Introduction

Random-pattern skin flap is a technique used in tissue reconstruction, and is not limited in flap position and direction due to the lack of axial vasculature^1,2^. Therefore, this technique is particularly popular in various clinical specialties such as plastic, trauma, and hand surgery^3,4^. However, due to the lack of axial blood vessels, the skin flap’s blood supply mainly depends on the microvascular network at the pedicle of the flap, and the blood flow at the distal end of the flap is often poor and inadequate, often leading to ischemic necrosis^5,6^. Ischemia is a particularly troublesome issue when the length-to-width ratio of the flap exceeds 2:1, greatly limiting the clinical application and efficacy of the random flap^7,8^. Thus, novel strategies to improve flap viability are of great clinical and scientific interest.

Various published studies have used growth factors to augment skin flap survivability, especially fibroblast growth factors (FGF)^9–11^. For example, FGF1 and FGF2 were shown to improve survival of ischemic skin flap through increasing cutaneous vasculature and preventing ischemia^10–12^. In 2000, Nishimura et al. isolated fibroblast growth factor 21 (FGF21) from mouse embryonic tissues^13^, which regulated various metabolic functions^14,15^. Since its discovery, FGF21 has been reported to normalize glucose and lipid homeostasis, thus preventing the development of metabolic disorders, such as obesity and diabetes^16,17^. Furthermore, FGF21 is also found to exert cell-protective effects in metabolically active organs, such as the liver and pancreas^18,19^. Recently, FGF21 has been shown to promote angiogenesis, inhibit oxidative stress and apoptosis in vascular diseases^20–22^. As vascular networks from the pedicle of random skin flaps is often insufficient to supply blood and nutrients to the distal flap, angiogenesis is thought to play a critical role for the survival of distal flaps^1,23^. Furthermore, reducing oxidative stress can also help improve skin flap viability by limiting ischemia-reperfusion injury in ischemic tissues when blood flow is recanalized^24–26^. Thus, we hypothesized that FGF21 can promote the survival of random flaps by promoting angiogenesis and inhibiting oxidative stress.

In addition to angiogenesis and oxidative stress, autophagy, a lysosomal-dependent and highly conserved process of macromolecular material circulation in eukaryotic cells, is also essential for cell survival and maintenance^27^. Past reports have suggested that FGF21 could promote autophagy in several models^28–30^, and that FGF21’s protective effects in ischemic and ischemia-reperfusion injuries are due to its ability to upregulate autophagy^31^. However, while autophagy may enhance cell survival, it can also accelerate cell death. Therefore, it is unclear if FGF21’s modulation of autophagy will be beneficial in all settings of ischemia, and there have been no past studies of the effect of FGF21 in the random flap model.

In this study, we explored whether FGF21 plays a substantial role in modulating the viability of random skin flaps by evaluating its effects on angiogenesis, oxidative stress, and autophagy. Furthermore, using conventional molecular biology methods, we performed mechanistic studies to elucidate the mechanism by which FGF21 improves survival of skin flaps. Overall, our results suggest that FGF21 significantly improves skin flap viability via activation of TFEB through AMPK signaling pathways, which leads to increased autophagy, subsequently upregulating angiogenesis and reducing oxidative stress.

## Materials and methods

### Animals and ethics statement

Adult male Sprague-Dawley rats (250–300 g) from Wenzhou Medical University (License No. SCXK [ZJ] 2005-0019), were housed in a standard condition (temperature: 23 ± 2 °C, humidity: 50 ± 5%, 12 h light/dark cycle), and free fed with food and water. Experimental procedures involving animals complied with the Guide for Care and Use of Laboratory Animals of the China National Institutes of Health, with acceptance of the Animal Care and Use Committee of Wenzhou Medical University (wydw 2017-0022).

### Antibodies and regents

These following chemicals were used in the study: FGF21 (P6101) was obtained from Beyotime Biotechnology (Jiangsu, China); 3-methyladenine (3MA, M9281) from Sigma-Aldrich Chemical Company (Milwaukee, WI, USA); Dorsomorphin (Compound C, HY-13418A) and Torin1 (HY-13003) were purchased from Med Chem Express (Monmouth Junction, NJ, USA). H&E Staining Kit, DAB developer from Solarbio Science & Technology (Beijing, China); the BCA Kit, NE-PER™ Nuclear and Cytoplasmic Extraction Reagents, and Immunoprecipitation Kit from Thermo Fisher Scientific (Rockford, IL, USA); the ECL Plus Reagent Kit from PerkinElmer Life Sciences (Waltham, MA, USA); superoxide dismutase (SOD), glutathione (GSH), malondialdehyde (MDA) assay kits, and pentobarbital sodium from Jiancheng Technology (Nanjing, China). Primary antibodies were purchased from companies as following: FGF21 (26272-1-AP), VEGF (19003-1), SOD1 (10269-1), VPS34 (12452-1), MMP9 (10375-2), HO1 (10701-1), cathepsin D (CTSD, 21327-1), and Caspase 3 (CAPS3, 19677-1) from Proteintech Group (Chicago, IL, USA); cytochrome c (CYC, 14796), Bax (32027), endothelial nitric oxide synthase (eNOS, 11940S), AMPK (2532), p-AMPK (2537), mTOR (2983), p-mTOR (2971), FOXO3a (12829), p-FOXO3a (9466), and CARM1 (3379) from Cell Signaling Technology (Beverly, MA, USA); P-TFEB (Ser221) (AF3708) from Affinity Biosciences (OH, USA), SQSTM1/p62 (ab56416) from Abcam (Cambridge, UK); LC3B (L7543) from Sigma-Aldrich Chemical Company (Milwaukee, WI, USA); Cadherin5 (A02632-2) from Boster Biological Technology (Wuhan, China); GAPDH (AP0063) from Biogot Technology (Shanghai, China); Histone-H3 (17168-1-AP) and SKP2 (15010-1-AP) from Proteintech Group (Chicago, IL, USA). Horseradish peroxidase (HRP)-conjugated IgG secondary antibody was purchased from Santa Cruz Biotechnology (Dallas, TX, USA). Fluorescein isothiocyanate (FITC)-conjugated IgG secondary antibody was obtained from Boyun Biotechnology (Nanjing, China). And 4′,6-Diamidino-2-phenylindole (DAPI) solution was from Beyotime Biotechnology (Jiangsu, China).

### Establishment of a skin flap model

Two percent (w/v) of pentobarbital sodium (40 mg/kg) was injected intraperitoneally to rats for anesthesia. The random-pattern skin flap was established in the central dorsum according to the modified McFarlane flap model performed in published studies^32,33^. In brief, after shaving hair and disinfecting, a caudal skin flap (3 cm × 9 cm) was cut and separated from subcutaneous deep fascia on the rat back. Subsequently, both sacral arteries were exposed and sectioned. Then the flap was covered on the donor bed and sutured by 4-0 silk. The flap was averagely divided into three area from its distal part to the pedicle in the following order: Area I, Area II, and Area III. Sham rats did not received any operation, and the skin in the dorsum back was marked to delineate Area I, Area II, and Area III, corresponding to animals with flap surgery. In our experimental model, Area I is normally healthy, and Area III is normally necrotic without intervention. Area II, on the other hand, is usually ischemic and tending toward necrosis. To promote flap survival, Area II was used as the watershed area at which therapies are targeted to inhibit ischemia and potential necrosis. Therefore, Area II was selected for histological and molecular biological examination.

### Preparation of adeno-associated virus (AAV) vector

AAV-TFEB shRNA was obtained from Shanghai Genechem Company (Shanghai, China). pAV-U6-shRNA (TFEB)-CMV-EGFP was produced by synthesizing TFEB-activated protein kinase’s shRNA sequence and cloning into pAV-U6-shRNA-CMV-EGFP plasmid. Then, pAV-U6-shRNA (TFEB)-CMV-EGFP, Ad helper (adenovirus helper plasmid), and AAV Rep/Cap expression plasmid were used to produce AAV9-U6-shRNA (TFEB)-CMV-EGFP via transfection of AAV-293 cells. In a similar process, scramble control was produced using AAV9-U6-shRNA (scramble)-CMV-EGFP. Iodixanol gradient method was used to purify viral particles. Titers of AAV9-U6-shRNA (TFEB)-CMV-EGFP and AAV9-U6-shRNA (scramble)-CMV-EGFP were determined by quantitative PCR, and were 1.243 × 10^12^, 1.22 × 10^12^ genomic copies per ml, respectively.

### Animal groups and treatment protocols

Hundred and forty-four rats operated with random-pattern skin flap were randomly divided into seven groups: a Control group (*n* = 30), a FGF21 group (*n* = 30), a FGF21 + 3MA group (*n* = 24), a FGF21 + AAV-Scramble Control group (Scramble, *n* = 18), a FGF21 + AAV-TFEB short hairpin RNA group (TFEB shRNA, *n* = 18), a FGF21 + CC group (*n* = 12), a FGF21 + CC + Torin1 group (*n* = 12). The FGF21 group was treated with FGF21 (100 μg/kg/day) by daily subcutaneous injection for 7 days after operation of flap model, while the Control group with equal volume of saline using the same protocol. The FGF21 + 3MA mice were treated with FGF21 (100 μg/kg/day) and 3MA (0.5 mg/kg/day) with the same protocol. The FGF21 + CC group and FGF21 + CC + Torin1 groups received CC (1.5 mg/kg/day, intraperitoneal) or Torin1 (2 mg/kg/day, intraperitoneal) with the same dose of CC 30 min before FGF21 administration (dose) every time. Fourteen days before the operation, rats in the Scramble group and the TFEB shRNA group received 18 micro ml viral vectors daily in PBS with 5 × 10^9^ packaged genomic particles total by subcutaneous injections via a microsyringe; after the surgical procedure, AAV-treated rats received FGF21 using the aforementioned protocol. Seven days after the surgical procedure, all animals were euthanized with excess anesthesia, and tissue samples (0.5 cm × 0.5 cm) were harvested from Area II of the flaps.

### Assessment of flap survival area and edema

Macroscopic behavior of the flap was observed and recorded daily. The survival area percentage of skin flap was assessed using photograph of flap by ImageJ software (National Institutes of Health, Bethesda, MD): The percentage of survival area was calculated with the following formula: % survival = survival area ÷ total area × 100%.

Flap tissue edema was evaluated by the tissue water content. Samples harvested on post-op day (POD) 7 were dehydrated in an autoclave of 50 °C and weighed daily until the weight remained constant over 2 days. Then the tissue water content was calculated as: percentage of tissue water content of flap = (weight prior to autoclave − final dry weight) ÷ initial weight × 100%.

### Laser Doppler blood flow (LDBF) imaging

Blood supply under flap was visualized using LDBF imaging. Rats were anesthetized and placed in a prone position on the 7th day after surgery, and the skin flap was scanned by Laserflo BPM (Vasamedic, St. Paul, MN, USA), a laser doppler instrument that features deep penetration and ability to visualize small vessels with adequate depth^34^. The results were quantified by moor LDI Review software (ver.6.1; Moor Instruments), and blood flow intensity was assessed using perfusion units (PU). Scans were repeated three times and averaged for each animal before statistical comparison between groups.

### Hematoxylin and eosin (H&E) staining

Six tissue samples were collected per group for histopathologic analysis. Specimen were fixed in 4% (v/v) paraformaldehyde and embedded in paraffin wax before being cut as tissue sections of 4-μm thickness, which were used for H&E staining. Next sections were visualized via light microscopy (×200 magnification) to evaluate histological changes, including swelling, granulation, and microvascular reconstruction. To assess the level of microcirculation, mean vessel density of flap tissue was counted by the number of vascular cross section per unit area (/mm^2^) from randomly selected fields.

### Immunohistochemistry (IHC)

After deparaffinizing tissue specimen in xylene, ethanol was used for rehydration, and samples were blocked with 3% bovine serum and repaired in 10.2 mM sodium citrate buffer. Then, the following primary antibodies were used for incubation overnight as 4 °C: CD34 (1:100), Cadherin5 (1:100), VEGF (1:300), CASP3 (1:200), SOD1 (1:100), CTSD (1:100). Then, samples were treated with HRP-conjugated secondary antibody, stained by DAB kit, and counterstained with hematoxylin. Lastly, stained sections were visualized under light microscopy (×200 magnification) using the DP2-TWAN image-acquisition system (Olympus Corp., Tokyo, Japan). Quantification was performed for Cadherin5, VEGF, SOD1, CASP3, and CTSD expression levels, and number of CD34-positive blood vessels was enumerated. Measurements were obtained from three random sections, with six random visual fields each.

### Immunofluorescence

Tissue specimen were deparaffinized and rehydrated as described above. Three percent (v/v) of H_2_O_2_ was used to quench endogenous peroxidase and tissue antigen was repaired with 10.2 mM sodium citrate buffer. Three percent bovine serum for 30 min was used for blocking. Specimen were incubated with primary antibodies against LC3B (1:200), TFEB (1:100) for 2 h at room temperature for primary staining, followed by 1 h, 37 °C incubation with anti-rabbit secondary antibodies DAPI staining. Cells from the specimen were visualized using fluorescence microscope and the DP2-TWAN image-acquisition system (Olympus Corp., Tokyo, Japan). This method was used to assess LC3II-positivity and TFEB translocation into nucleus.

### TUNEL staining

DNA damage was assessed using TUNEL (terminal deoxynucleotide transferase-mediated (dUTP) nick-end labeling) staining according to manufacturer’s instructions. In brief, sections, after deparaffinization, rehydration, blocking, and treatment with 10.2 mM sodium citrate buffer, were stained for 30 min at 37 °C with in situ cell death detection kit (Roche China, Shanghai, China). Then, nuclei was stained with DAPI. TUNEL positive cells were visualized using a fluorescence microscope (Olympus Inc., Tokyo, Japan), enumerated in six randomly selected fields from each specimen.

### Western blotting and Immunoprecipitation

After animals were euthanized, skin samples from Area II of flap were harvested for western blotting and Immunoprecipitation. For western blotting, six samples per group were homogenized in radioimmunoprecipitation assay (RIPA) lysis buffer (#sc-24948, Santa Cruz Biotechnology, Inc., Dallas, Texas), yielding lysates of total tissue protein. Another six samples per group (except the FGF21 + 3MA group) were processed to extract cytoplasmic protein and nuclear protein by NE-PER (Nuclear and Cytoplasmic Extraction Reagents). Then, protein contents were measured with BCA assay, and protein was separated by sodium dodecyl sulfate polyacrylamide gel electrophoresis (SDS-PAGE), electro-transferred to polyvinylidene difluoride (PVDF) membranes, blocked with 5% skimmed milk, and probed with the following antibodies overnight at 4 °C: FGF21(1:1000), VEGF (1:1000), MMP9 (1:1000), Cadherin5 (1:1000), CYC (1:1000), Bax (1:1000), CASP3 (1:1000), SOD1 (1:1000), eNOS (1:1000), HO1 (1:1000), Beclin1 (1:1000), VPS34 (1:1000), LC3B (1:500), CTSD (1:1000), p62 (1:1000), TFEB (1:1000); P-TFEB (Ser221) (1:1000), AMPK (1:1000), p-AMPK (1:1000), FOXO3a (1:1000), p-FOXO3a (1:1000), CARM1 (1:1000), Histone-H3 (1:1000), SKP2 (1:1000), and GAPDH (1:1000). After 2 h secondary antibody incubation, the ECL Plus Reagent Kit was used to visualize bands. Image Lab 3.0 software (Bio-Rad, Hercules, CA, USA) was used to measure intensity of each band. For Immunoprecipitation studies, briefly, antibodies were immobilized to the Coupling Resin, and tissue lysates were pre-cleaned and immunoprecipitated with the immobilized resin at 4 °C overnight. Samples were eluted for western blotting. All visual WB results are presented as a pair of marker and GAPDH bands from one sample most representative of the group.

### Assessment of SOD, GSH, and MDA level

Oxidative stress levels in ischemic skin flaps were evaluated using assays for SOD, GSH and MDA. Seven days after operation, specimen from Area II was stored at −80 °C, and then weighed, homogenized, and diluted to 10% (v/v) in ice bath. Then, SOD activity was assessed by the xanthine oxidase method, GSH level by modified 5,5′-dithiobis [2-nitrobenzoic acid] method, and MDA content by reaction with thiobarbituric acid (TBA) at 90–100 °C.

### Statistical analysis

Statistical analysis was performed by SPSS ver. 19 software (Chicago, IL, USA). All data are expressed as means ± SEM. Comparisons between two independent groups were performed using two-tailed, unpaired *t*-test. Comparisons among more than three groups was performed using one-way ANOVA, with Bonferroni adjustment for post hoc pairwise comparisons. A *p*-value <0.05 was considered statistically significant.

## Results

### FGF21 ameliorates random-pattern skin flap survival

**Fig. 1:**
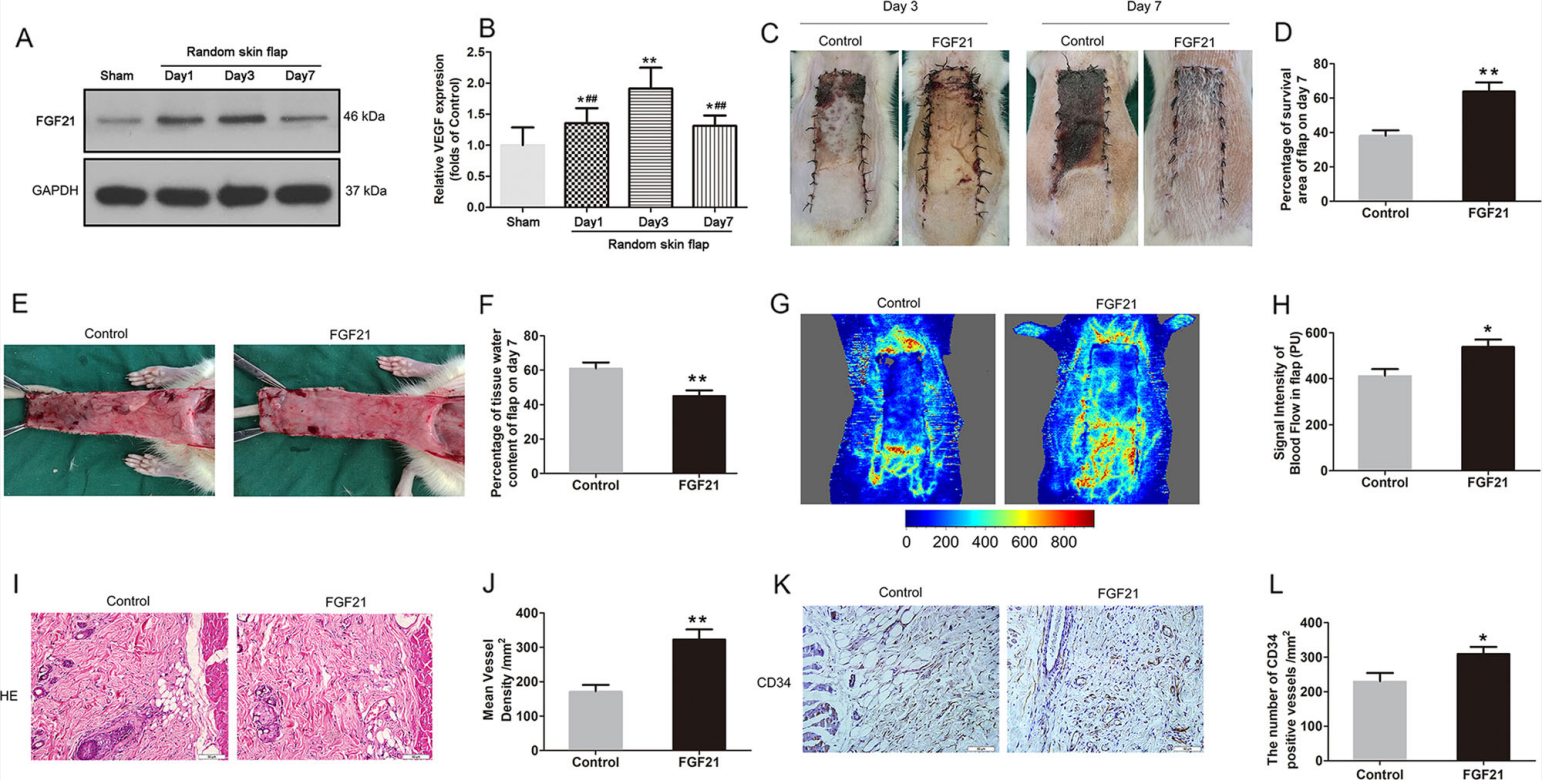
FGF21 ameliorates random-pattern skin flap survival. FGF21 level was evaluated after the establishment of rat random skin flap model without drug administration. On Day 7, animals were euthanized, and then the flap survival area and subcutaneous microcirculation were evaluated. **a** Western blot showing FGF21 levels at several time points after the operation of random skin flap. **b** Histogram showing quantification of the FGF21 expression assessed by western blotting. **c** Digital photograph of flap survival/necrosis area on the 3rd and 7th day after surgery. **d** Histogram showing the percentage of survival area of flap on the 7th day. **e** Digital photograph exhibiting tissue necrosis, edema and the subcutaneous vascular network on the inner surface of the flap. **f** Histogram showing the percentage of tissue water content of flap. **g** Subcutaneous vascular network detected by LDBF, a stronger signal intensity representing greater blood flow. **h** Histogram showing signal intensity of blood flow in flap. **i** H&E staining exhibiting subcutaneous histology of the flap, showing subcutaneous blood vessels and inflammation (original magnification × 200; scan bar, 50 μm). **j** Histogram showing mean vessel density (MVD) in the flap (/mm^2^). **k** IHC of CD34 which are mainly expressed in vascular endothelial cells; **l** Histogram showing the density of CD34-positive blood vessels (/mm^2^). Significance: *p < 0.05 and **p < 0.01 vs. Control group or Sham group; ^#^*p* < 0.05 and ^##^*p* < 0.01 vs. random skin flap Day 3 group. Data were expressed as means ± SEM (*n* = 6 per group).

After the “modified McFarlane flap model” was performed in rats, the distal part of flaps became swollen and pale. Necrosis developed in flap Area I, with the tissue appearing dark, dry, crumpled, and stiff. Necrosis gradually expanded toward the pedicle of skin flaps and its progression subsided on Day 7 after flap establishment. In order to detect the expression of FGF21 after random skin flap establishment, we excised tissue from the center part of Area II at several time points from the operation, and FGF21 levels were assessed. Western blot analyses showed that FGF21 was significantly enhanced in skin flaps immediately after establishment compared with the Sham group, with levels peaking at Day 3 (Fig. 1a, b). Therefore, we hypothesized that FGF21 may play a substantial role in the survival of random skin flaps. To further explore the relationship of FGF21 and skin flap survival, we administered exogenous FGF21 and compared flap survival at 7 days compared with flaps in the control group. Here, we found significantly improved flap survival in the group treated with FGF21 compared with the Control group (Fig. 1c, d). Qualitatively, flaps in the Control group were more swollen and bruised with more obvious venous blood stasis (Fig. 1e). Water content of flap, which is a measure of tissue edema, was significantly higher in the Control group than the FGF21 group (Fig. 1f).

Microvascular network reconstruction was visualized by LDBF (Fig. 1g). The FGF21 group showed a significantly stronger signal intensity of blood flow in flaps than the Control group (Fig. 1h). Vessel density was also evaluated using histology staining with H&E (Fig. 1i), and vessel density in the FGF21 group was significantly increased compared with controls (Fig. 1j). Similarly, the number of CD34-positive vessels under IHC was also significantly higher in the FG21 group than in the control group (Fig. 1k, l). Together, these results show that FGF21 plays a crucial role in flap survivability, and that exogenous administration of FGF21 leads to significantly improved flap survival by preventing ischemia.

### FGF21 upregulates angiogenesis in flaps

**Fig. 2:**
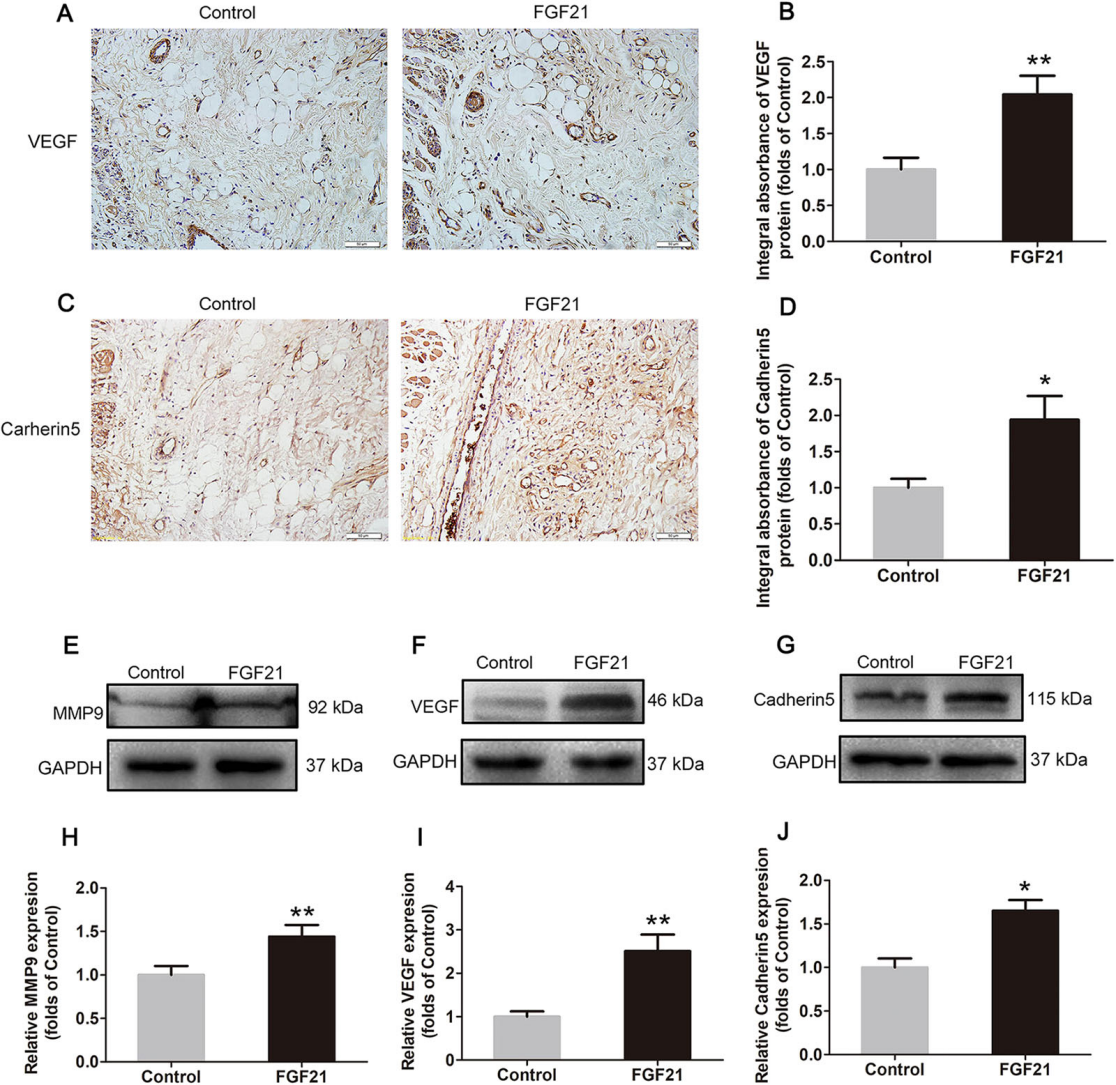
FGF21 upregulates angiogenesis in flaps. On the 7th day after operation, the samples were harvested for the evaluation of angiogenesis in ischemic skin flaps. **a**, **c** IHC of proteins VEGF and Cadherin5 in ischemic skin flaps (original magnification, ×200; scan bar, 50 μm). **b**, **d** Quantification of integral absorbance of VEGF and Cadherin5 in IHC. **e**, **f**, **g** Western blotting performed for expression of MMP9, VEGF, and Cadherin5, which was corrected by GAPDH as internal control. **h**, **i**, **j** Quantization of optical density values of MMP9, VEGF and Cadherin5 expressions in the flaps. Significance: *p < 0.05 and **p < 0.01 vs. Control group. Data were expressed as means ± SEM (*n* = 6 per group).

To explore whether FGF21 upregulates angiogenesis in skin flaps, we measured the levels of various markers of angiogenesis using IHC and western blotting. Results from both methods showed that VEGF, which is expressed in vascular endothelial cells and stromal cells (Fig. 2a), increased significantly in the FGF21 group compared with the Control group (Fig. 2b, f, i). Likewise, integral absorbance of Cadherin5 in IHC and its expression in western blotting were increased by FGF21 treatment (Fig. 2c, d, g, j). Furthermore, FGF21 enhanced the level of MMP9 as well (Fig. 2e, h). These results suggest that FGF21 upregulates angiogenesis, a key determinant of skin flap survival, by activating the expression of VEGF, MMP9, and Cadherin5.

### FGF21 inhibits apoptosis in flaps

**Fig. 3:**
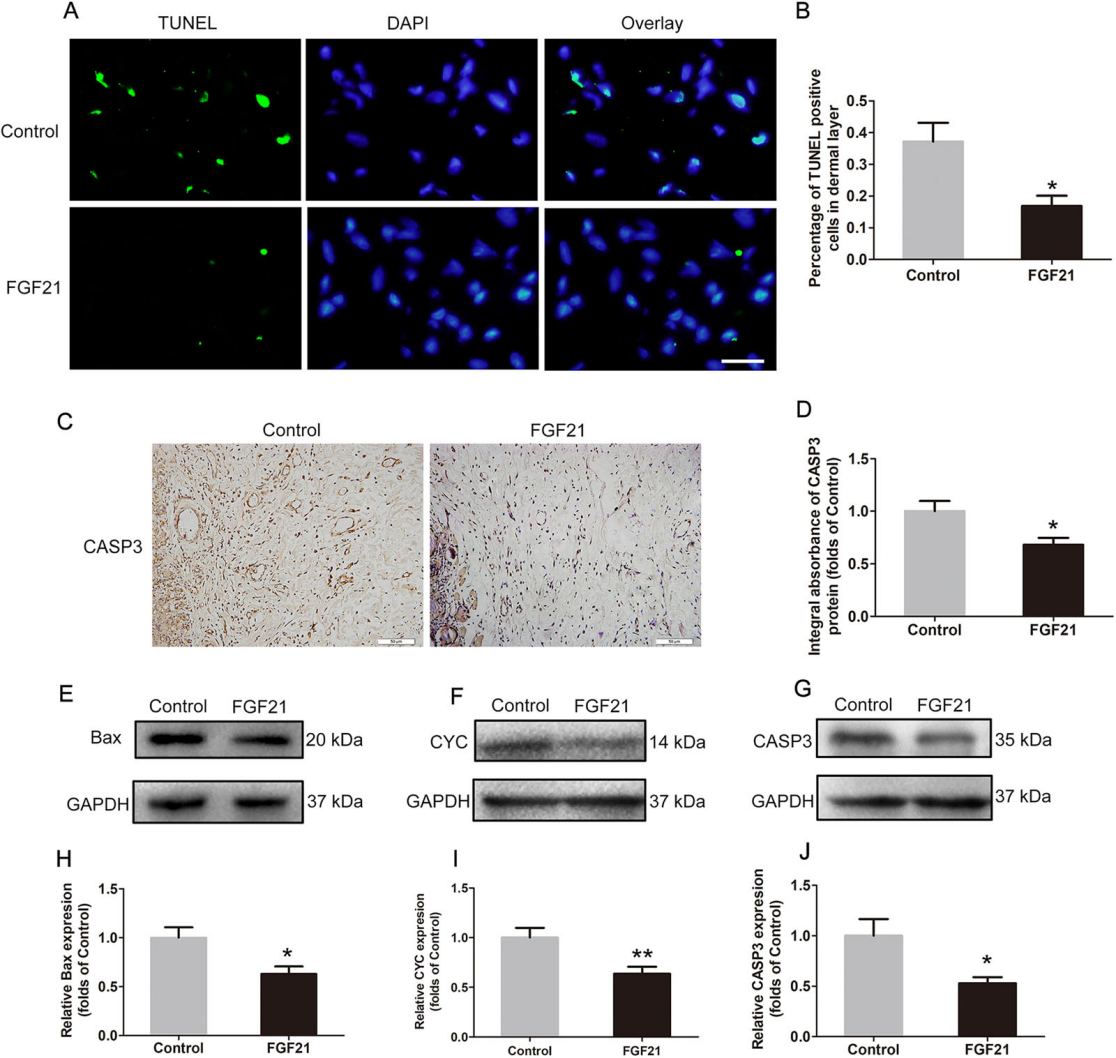
FGF21 inhibites apoptosis in flaps. **a** TUNEL assay performed to assess the level of DNA damage in the ischemic random skin flaps (scan bar, 15 μm) (nuclei, blue; damaged DNA, green). **b** Quantification of percentage of TUNEL positive cells in dermal layer. **c** Expression of CASP3 detected by IHC in flaps. **d** Quantification of integral absorbance of CASP3 protein. **e**, **f**, **g** Western blotting performed for expression of Bax, CYC, CASP3, which was corrected by GAPDH as internal control. **h**, **i**, **j** Quantization of optical density values of Bax, CYC, CASP3 expression in the flaps. Significance: *p < 0.05 and **p < 0.01 vs. Control group. Data were expressed as means ± SEM (*n* = 6 per group).

To explore possible mechanisms of cell-death inhibition by FGF21, we assessed DNA damage by TUNEL staining, in which the nuclei were stained blue and dUTP nick-ends of damaged DNA were labeled green. As shown, levels of DNA damage were decreased in the FGF21 group (Fig. 3a, b). This finding suggests that levels of apoptosis, a form of programmed cell death, may also play a role in modulating flap survival in FGF21-treated animals. To assess the levels of apoptotic activity, IHC and western blotting were used to analyze apoptosis-related proteins. Under IHC (Fig. 3c), decreased integral absorbance of CASP3 was observed in the FGF21 group (Fig. 3d). Moreover, levels of Bax, CYC and CASP3 were analyzed by western blotting (Fig. 3e, f, g), showing significantly lower expressions in the FGF21 group (Fig. 3h, i, j). Together, these results indicate that FGF21’s positive effect on flap survival may be due to inhibition of apoptosis and subsequent DNA damage.

### FGF21 attenuates oxidative stress in flaps

**Fig. 4:**
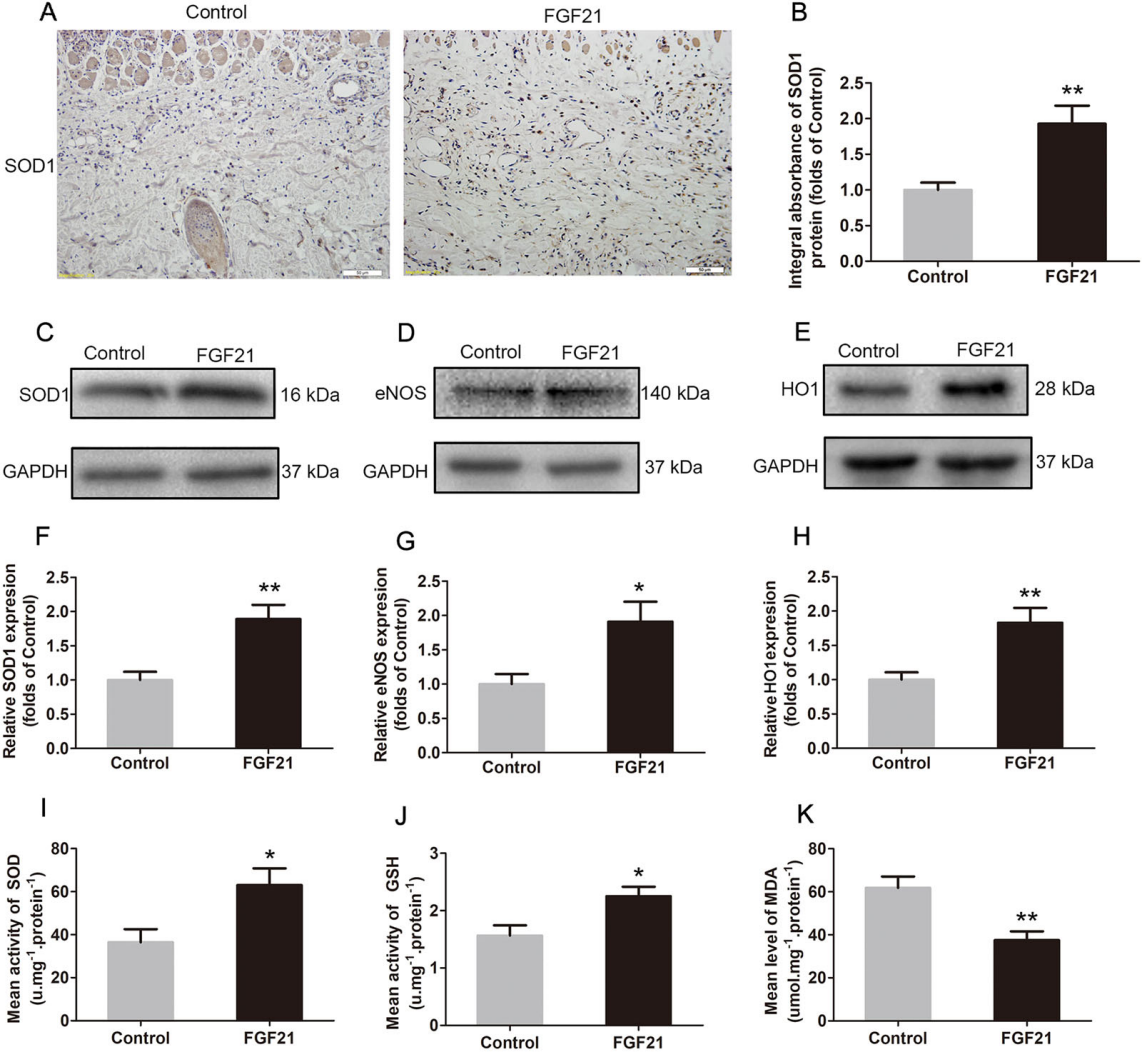
FGF21 attenuates oxidative stress in flaps. On the 7th day after operation, the samples were harvested for the evaluation of oxidative stress in ischemic skin flaps. **a** IHC of protein SOD1 in ischemic skin flaps (original magnification, × 200; scan bar, 50 μm). **b** Quantification of integral absorbance of SOD1 in IHC. **c**, **d**, **e** Western blotting performed for expression of SOD1, eNOS and HO1, which was corrected by GAPDH as internal control. **f**, **g**, **h** Quantization of optical density values of SOD1, eNOS, and HO1 expression. **i** Evaluation of SOD level using the assay of the xanthine oxidase method. **j** Evaluation of GSH level using modified 5,5′-dithiobis method. **k** Evaluation of MDA content using the modified TBA test. Significance: *p < 0.05 and **p < 0.01 vs. Control group. Data were expressed as means ± SEM (*n* = 6 per group).

Oxidative stress, and subsequent damage, is a key factor in skin flap necrosis, especially during perfusion-reperfusion injury. Thus, we assessed whether FGF21 impacts oxidative stress levels in skin flaps. IHC analysis showed that FGF21 effectively increased the level of SOD1, a crucial enzyme against oxidative stress (Fig. 4a, b). Western blotting also showed the similar results SOD1 levels in the FGF21 group (Fig. 4c, f). We also found higher levels of eNOS (Fig. 4d, g) and HO1 (Fig. 4e, h) in the FGF21 group, consistent with a lower level of oxidative stress. Furthermore, SOD, GSH, and MDA assays were processed, and results demonstrated that SOD activity (Fig. 4i) and GSH content (Fig. 4j), two endogenous antioxidative chemicals, increased significantly in the flap tissue with FGF21 treatment. MDA content, on the other hand, was depressed in the FGF21 group (Fig. 4k). Together, these results demonstrate that FGF21 leads to significantly reduced oxidative stress, possibly contributing to its pro-survival effects on random-pattern skin flaps.

### FGF21 promotes autophagy in flaps

**Fig. 5:**
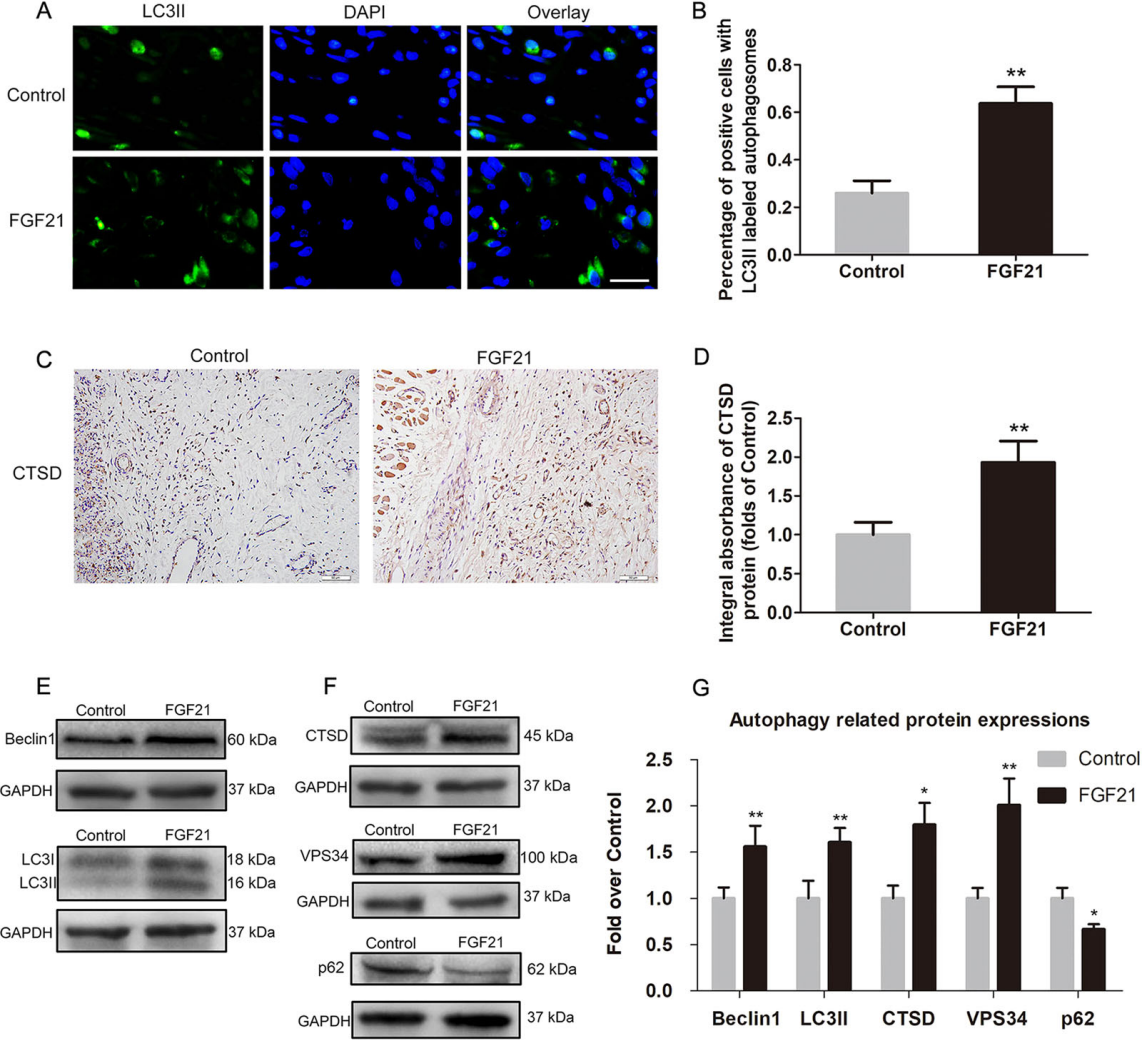
FGF21 augmentes autophagy in flaps. On the 7th day after operation, the samples were harvested for the evaluation of autophagy. **a** Immunofluorescence for the assessment of LC3II in the ischemic skin flap (scan bar, 15 μm). **b** Quantification of percentage of positive cells with LC3II labeled autophagosomes in dermal layer. (**c**) CTSD expression in ischemic skin flap evaluated by IHC. **d** Histogram showing expression of CTSD detected by IHC. **e**, **f** Western blotting for expression of Belin1, LC3II, CTSD, VPS34. and p62, which was corrected by GAPDH as internal control. **g** Histogram showing the levels of Belin1, LC3II, CTSD, VPS34, and p62 detected by western blotting. Significance: *p < 0.05 and **p < 0.01 vs. the Control group. Data were expressed as means ± SEM (*n* = 6 per group).

Given FGF21’s activity on angiogenesis, apoptosis, and oxidative stress, we hypothesized that FGF21’s effects may be due in part to modulation of autophagy. To this end, autophagy-related proteins were analyzed to make sure whether autophagy was activated by FGF21. Beclin1, BPS34 and LC3II are essential constituent proteins of autophagosomes, CTSD is a marker of lysosomes, and p62 is a substrate of autophagic flow^27^. Our results showed higher percentage of positive cells with LC3II labeled autophagosomes in the dermal layer of the FGF21 group than the Control group (Fig. 5a, b). By IHC, we found that FGF21 increased expression of CTSD (Fig. 5c, d). Moreover, western blot analysis revealed higher levels of Beclin1, as well as LC3II, CTSD, and VPS34, in flap tissues derived from the FGF21 group (Fig. 5e, f, g), with lower levels of p62. These results collectively suggest that FGF21 promotes autophagy in rat random-pattern skin flap.

### 3MA reverses effect of FGF21 on survival promotion in flaps

**Fig. 6:**
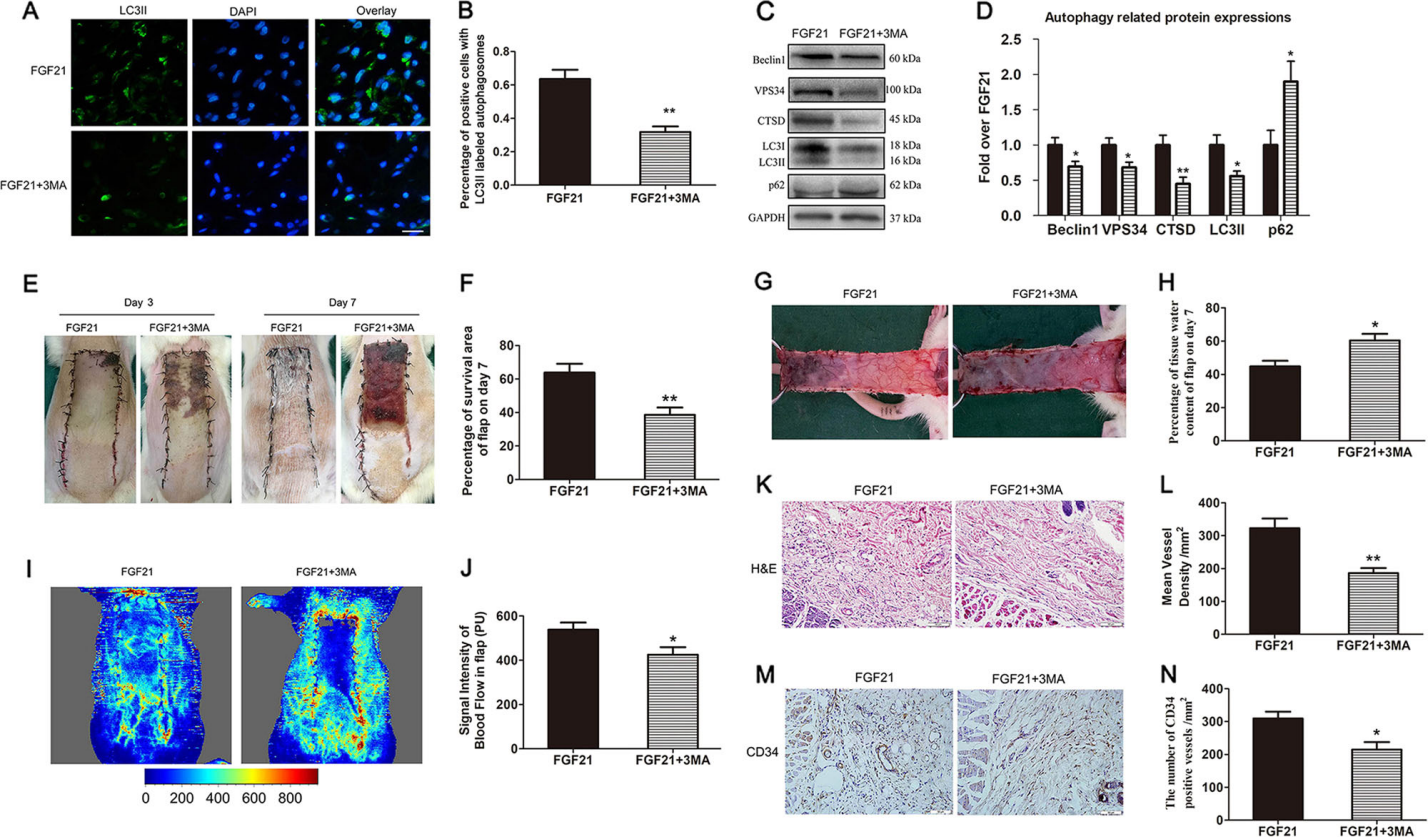
3MA reversed FGF21’s effect on random-pattern skin flap viability. On the 7th day after operation of random flap, the rats were sacrificed in the FGF21 group and FGF21 + 3MA group, and then autophagy level, survival area and subcutaneous microcirculation in the flap were evaluated. **a** Immunofluorescence for the evaluation of LC3II in the ischemic skin flap (scan bar, 15 μm). **b** Quantification of percentage of positive cells with LC3II labeled autophagosomes in dermal layer. **c**, **d** Western blotting performed for levels of autophagic proteins (Belin1, LC3II, CTSD, VPS34, and p62), which was corrected by GAPDH as internal control. **a** Histogram showing the quantification of autophagic proteins (Belin1, LC3II, CTSD, VPS34, and p62) detected by western blotting. **e** Digital photograph of flap survival/necrosis area on the 3rd and 7th day after surgery. **f** Histogram showing the percentage of survival area of flap on the 7th day. **g** Digital photograph exhibiting tissue necrosis, edema and the subcutaneous vascular network on the inner surface of the flap. **h** Histogram showing the percentage of tissue water content of flap. **i** The subcutaneous vascular network detected by LDBF, with stronger signal intensity representing greater blood flow. **j** Histogram showing signal intensity of blood flow in flap. **k** H&E staining exhibiting subcutaneous histology of the flap, showing subcutaneous blood vessels and inflammation (original magnification × 200; scan bar, 50 μm). **l** Histogram showing mean vessel density (MVD) in the flap (/mm^2^). **m** IHC of CD34 which mainly were expressed in vascular endothelial cells. **n** Histogram showing the density of CD34-positive blood vessels (/mm^2^). Significance: *p < 0.05 and **p < 0.01 vs the FGF21 group. Data were expressed as means ± SEM (*n* = 6 per group).

To determine whether FGF21’s increase of autophagy is beneficial for skin flap viability, we co-administered 3MA, a well-known autophagy inhibitor, with FGF21 and assessed outcomes. First, to validate that 3MA indeed inhibits autophagy when co-administered with FGF21, we assessed autophagy markers using IHC and western blotting. Immunofluorescence showed that the percentage of positive cells with LC3II labeled autophagosomes in the dermal layer was significantly reduced by 3MA (Fig. 6a, b), and WB also exhibited that 3MA co-administration significantly decreased the expression of LCII, Beclin1, VPS34, and CTSD, and obviously increased the expression of p62 (Fig. 6c, d). The above results demonstrated that 3MA successfully inhibited autophagy stimulated by FGF21 in random skin flaps.

Next, we evaluated whether 3MA co-administration impacted flap viability outcomes of FGF21-treated skin flaps. Our results showed that in the FGF21 + 3MA group, flap survival decreased significantly when compared with the FGF21 group (Fig. 6e, f). Similarly, 3MA worsened flap edema (Fig. 6g), resulting in a significant difference in water content (Fig. 6h). LDBF results also showed that 3MA reduced the density of subcutaneous blood vessels (Fig. 6i), and after quantification, the difference was significant (Fig. 6j). H&E staining revealed that the mean vascular density in the FGF21 + 3MA group was significantly reduced (Fig. 6k, l). Moreover, IHC staining showed that significantly fewer CD34-positive blood vessels were found in the FGF21 + 3MA group (Fig. 6m, n). Together, these results showed that 3MA largely reversed FGF21’s survival benefit for skin flaps. Thus, it could be speculated that activating autophagy is a main mechanism by which FGF21 promotes flap survival.

### 3MA reverses effects of FGF21 on angiogenesis, oxidative stress, and apoptosis in flaps

To further validate autophagy as a main player in FGF21’s benefit for skin flap survival, we explored the effect of 3MA co-administration on angiogenesis, oxidative stress, and apoptosis. Here, we found that the expression of angiogenesis-related proteins (MMP9, VEGF, Cadherin5) in the FGF21 + 3MA group was significantly reduced, comparing to the FGF21 group, indicating that autophagy promoted angiogenesis in the random flap model (Fig. SA, D). Similarly, 3MA significantly increased the expression of apoptosis-related proteins (Bax, CYC, CASP3), indicating that autophagy inhibited apoptosis in FGF21-treated skin flaps (Fig. SB, D). Also, 3MA significantly reduced the levels of protective proteins (SOD1, eNOS, HO1) against oxidative stress (Fig. SC, D). In addition, the tissue content of SOD (Fig. SE) and GSH (Fig. SF) was significantly less in the FGF21 + 3MA group, and MDA was increased (Fig. SG), indicating that autophagy protected tissues from oxidative stress in FGF21-treated flaps. Altogether, our results suggest that FGF21 increased the level of autophagy, which is a main mechanism by which FGF21 promotes angiogenesis, inhibits apoptosis and reduces oxidative stress in random-pattern skin flaps, ultimately improving flap viability.

### FGF21 boosted autophagy via enhancing TFEB activity

**Fig. 7:**
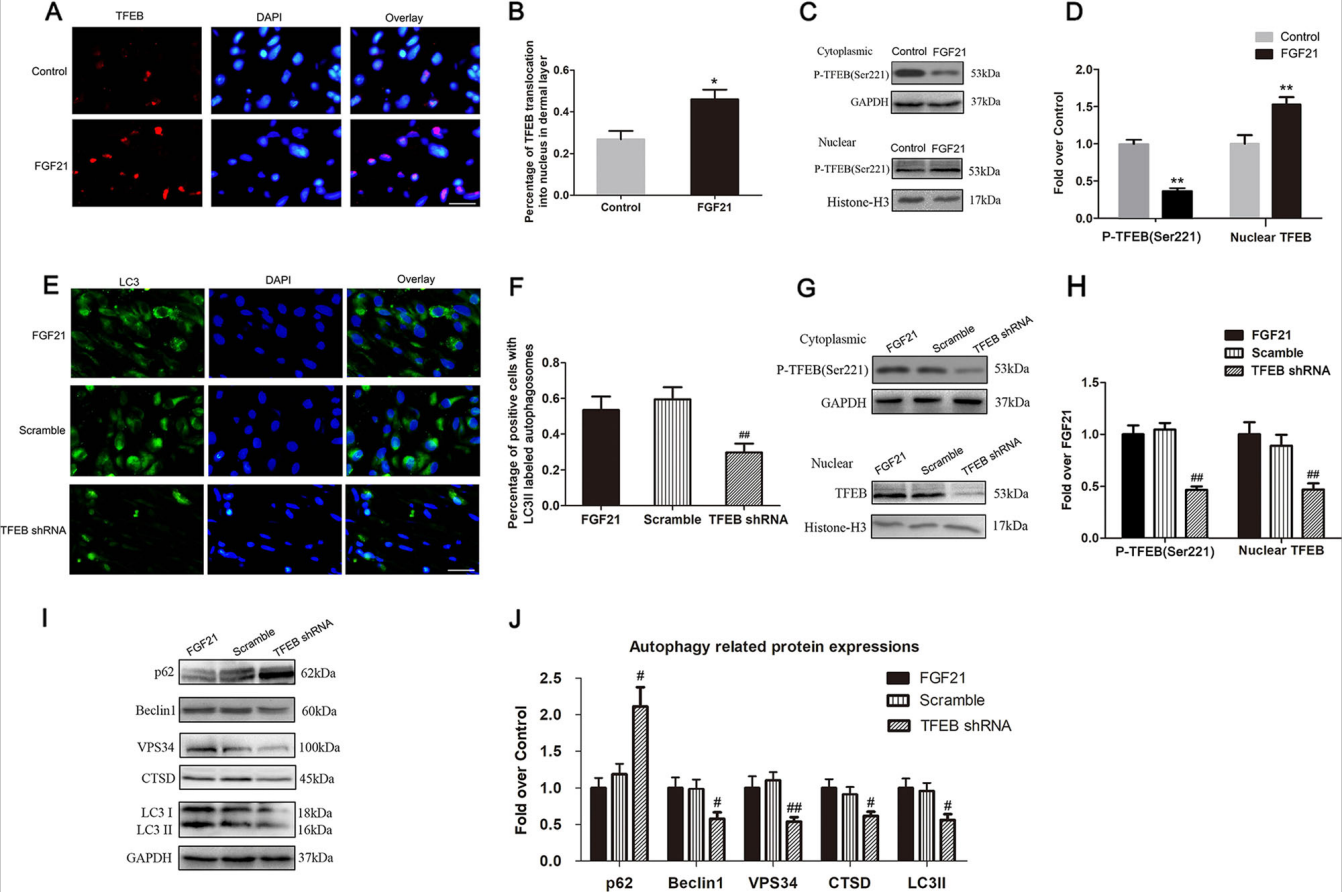
FGF21 boosted autophagy via enhancing TFEB activity. On the 7th day after operation, samples were harvested from the Control and FGF21 groups to evaluate the TFEB level. Further, transfection of the AVV TFEB shRNA was performed to inhibit the expression of TFEB in the cells, and then the expression of relative proteins was compared in the FGF21, Scramble and TFEB shRNA group. **a** Immunofluorescence exhibiting more expression of TFEB in the FGF21 group than that in the Control group (scan bar, 15 μm). **b** Histogram showing the percentage of TFEB translocation into nucleus in dermal layer. **c** Western blotting showing levels of P-TFEB (Ser221) and nuclear TFEB. **d** Histogram showing quantificational comparison of cytoplasmic and nuclear TFEB expressions between the Control and FGF21 groups. **e** Immunofluorescence exhibiting the expression of LC3II in the FGF21, Scramble and TFEB shRNA groups. **f** Quantification of percentage of positive cells with LC3II labeled autophagosomes in dermal layer. **g** Western blotting showing levels of P-TFEB (Ser221) and nuclear TFEB in the FGF21, Scramble and TFEB shRNA groups. **h** Quantification of cytoplasmic and nuclear TFEB expressions detected by western blotting. **i** Western blotting showing levels of autophagic proteins (p62, Beclin1, VPS34, CTSD, and LC3II) in the FGF21, Scramble and TFEB shRNA groups, which was corrected by GAPDH as internal control. **j** Quantification of expression of autophagy-related proteins (p62, Beclin1, VPS34, CTSD, and LC3II) in western blotting. Significance: *p < 0.05 and **p < 0.01 vs the Control group. ^#^*p* < 0.05 and ^##^*p* < 0^.^01 vs the Scramble group^.^ Data were expressed as means ± SEM (*n* = 6 per group).

Previous studies have shown that TFEB plays an important role in the regulation of autophagy. We assessed levels of dephosphorylation and nuclear translocation of TFEB after FGF21 treatment in random skin flaps, and our results showed that the percentage of TFEB translocation into nucleus in the dermal layer was significantly higher in the FGF21 group than the control group (Fig. 7a, b). In addition, western blotting also revealed that the intranuclear level of TFEB was significantly increased by FGF21, while the level of P-TFEB (Ser221) was decreased (Fig. 7c, d). These results suggest that FGF21 increased TFEB dephosphorylation, and nuclear translocation, indicating that FGF21 augments autophagy via activation of TFEB. To further validate that TFEB activation is responsible for FGF21’s augmentation of autophagy, we utilized TFEB shRNA to silence TFEB activity, and designed a trial comparing three groups: the FGF21-only group, the FGF21 + Scramble group (non-active shRNA), and the FGF21 + TFEB shRNA group. Results showed that both the level of P-TFEB (Ser221) and nuclear expression of TFEB in the TFEB shRNA group were significantly lower than that in the Scramble group, while there was no significant difference in nuclear expression of TFEB between the FGF21 group and the FGF21 + Scramble group (Fig. 7g, h). These results suggest that transfection of TFEB shRNA successfully inhibited phosphorylation and nuclear translocation of TFEB.

Next, we evaluated the effect of inhibiting TFEB on FGF21-induced autophagy in skin flaps. Immunofluorescence displayed that there was no significant difference in the proportion of positive cells with LC3II labeled autophagosomes in the subcutaneous tissues between the FGF21 group and the FGF21 + Scramble group, while the proportion in the TFEB shRNA group was significantly lower than in the FGF21 + Scramble group (Fig. 7e, f). Similarly, WB results showed that the levels of Beclin1, VPS34, CTSD, LC3II, p62 were not significantly different between the FGF21 group and the FGF21 + Scramble group, and the expression of Beclin1, VPS34, CTSD, LC3II, and p62 was significantly lower in the FGF21 + TFEB shRNA group than in the FGF21 + Scramble group, while p62 was opposite (Fig. 7i, j). Together, these results suggest that TFEB activation and nuclear translocation is a major mechanism by which FGF21 increases the level of autophagy.

### FGF21 activated AMPK-mTOR pathway and AMPK-FoxO3a-SPK2-CARM1 signaling cascade in flaps

**Fig. 8:**
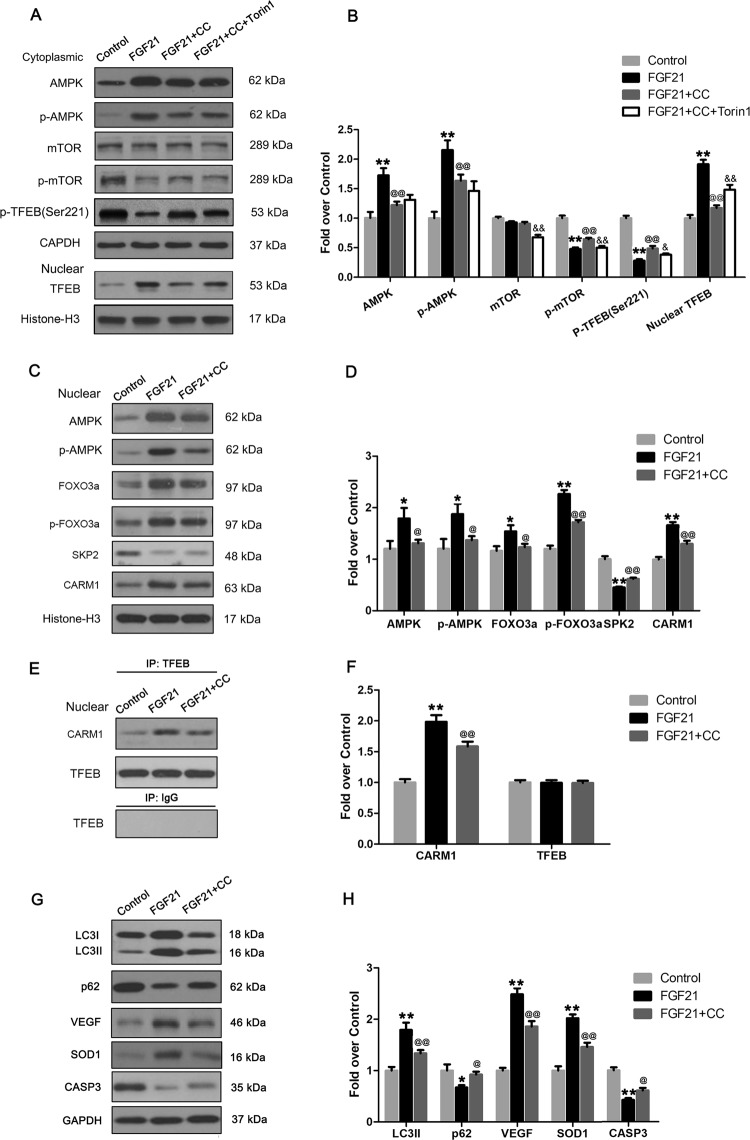
FGF21 activates AMPK-mTOR pathway and AMPK-FoxO3a-SPK2-CARM1 signaling cascade in flaps. On the 7th day after operation, samples were harvested from the Control, FGF21, FGF21 + CC, FGF21 + CC + Torin1 groups for the evaluation. **a** Western blotting showing the cytoplasmic levels of AMPK, p-AMPK, mTOR, p-mTOR and P-TFEB (Ser221) which were corrected by GAPDH as internal control; and nuclear levels of TFEB which were corrected by Histone-H3 as internal control. **b** Histogram showing quantificational comparison of AMPK, p-AMPK, mTOR and p-mTOR. **c** Western blotting showing the nuclear levels of AMPK, p-AMPK, FOXO3a, p-FOXO3a, SKP2, and CARM1 which were corrected by Histone-H3 as internal control. **d** Histogram showing quantificational comparison of AMPK, p-AMPK, FOXO3a, p-FOXO3a, SKP2, and CARM1 between the Control and FGF21 groups. **e** Western blotting for Immunoprecipitation of CARM1 and TFEB. **f** Histogram exhibiting the quantification of CARM1 and TFEB levels under Immunoprecipitation. **g** Western blotting showing levels of proteins of LC3II, p62, VEGF, SOD1, and CASP3 which were corrected by GAPDH as internal control. **h** Histogram showing quantificational comparison of LC3II, p62, VEGF, SOD1, and CASP3. Significance: *p < 0.05 and **p < 0.01 vs the Control group; ^@^*p* < 0.05 and ^@@^*p* < 0^.^01 vs the FGF21; ^&^*p* < 0^.^05 and ^&&^*p* < 0.01 vs the FGF21 + CC. Data were expressed as means ± SEM (*n* = 6 per group).

According to published reports, there are two important pathways that modulate TFEB: the AMPK-mTOR and the AMPK-FoxO3a-SPK2-CARM1 signaling cascades. We investigated whether these two pathways were activated in skin flaps treated with FGF21. Our results showed that FGF21 increased the expression of p-AMPK and inhibited p-mTOR in cytoplasm, while the expression of AMPK and mTOR was not significantly different in the two groups (Fig. 8a, b), indicating that FGF21 activated the AMPK-mTOR pathway. In the nucleus, FGF21 increased the expression of AMPK, p-AMPK, FOXO3a, p-FOXO3a, CARM1, and inhibited the expression of SPK2 (Fig. 8c, d), indicating that FGF21 also activated the AMPK-FOXO3a-SPK2-CARM1 pathway. To confirm the interaction of CARM1 and TFEB, Immunoprecipitation was performed in the Control and FGF21 groups. The result showed that the higher level of CARM1 associated with TFEB expression with consistency (Fig. 8e, f).

To investigate whether FGF21-induced TFEB activation was mediated by AMPK-FoxO3a-SPK2-CARM1 and AMPK-mTOR signaling pathways in skin flaps, we further explored the effects of compound C, an AMPK blocker, on AMPK-FoxO3a-SPK2-CARM1 and AMPK-mTOR signaling pathways and flap survival. Our results showed that FGF21 promoted the AMPK-mTOR pathway mediated TFEB dephosphorylation and nuclear translocation, and that these effects were reversed after use of compound C (Fig. 8a, b). The mTOR inhibitor, Torin1, was also used to examine whether the effect of FGF21 is through mTOR. Western blotting results indicated the mTOR activation and TFEB inhibition by compound C in FGF21-treated flaps were reversed after Torin1 treatment (Fig. 8a, b). Moreover, results of western blotting and Immunoprecipitation demonstrated that FGF21 activated AMPK-FoxO3a-SPK2-CARM1 signaling pathway in flaps, and compound C inhibited the FGF21-mediated signaling pathway stimulation (Fig. 8c–f). Finally, western blotting results also indicated compound C significantly inhibited FGF21-mediated enhancement of autophagy and angiogenesis, and depression of apoptosis and oxidative stress in the ischemic flaps (Fig. 8g, h). Together, our results confirmed that FGF21 activated TFEB in skin flaps through AMPK-FoxO3a-SPK2-CARM1 and AMPK-mTOR signaling pathways.

## Discussion

While random-pattern skin flaps are convenient tools in tissue reconstruction, distal ischemic necrosis is a common clinical complication, limiting the use of random flaps and reducing the success of surgery^35,36^. The main reasons for ischemic necrosis at the distal flap are insufficient blood supply and ischemia-reperfusion injury^36,37^. Oxidative stress and apoptosis are two important mechanisms of ischemia-reperfusion injury, leading to further damage or necrosis of the tissue^38,39^. In our studies, our results showed that FGF21 plays a central role in skin flap survival, and that administration of exogenous FGF21 improves flap viability by activation of autophagy and subsequent enhancement of angiogenesis, inhibition of apoptosis, and reduction of oxidative stress.

FGF21, a member of the FGF family, is a polypeptide of 209 amino acids, which has the function of regulating cell growth, differentiation and metabolism in the body^40^. Previous studies have shown that FGF21 promotes angiogenesis through Dynamin-2 and Rab5-mediated pathways in endothelial cell models^12^. Furthermore, FGF21 was reported to ameliorate myocardial ischemia/reperfusion injury through enhancing autophagy^31^. In addition, the role of FGF21 in inhibiting oxidative stress and apoptosis has also been reported^20,41^. Therefore, we hypothesized that FGF21 may reduce ischemic necrosis in random-pattern skin flaps, which was validated in our present work. Necrosis of the flap was fully evaluated, and the survival area, the water content, and the distribution of the vascular network under LDBF were all improved, suggesting that FGF21 is a potent promoter of flap viability.

To evaluate FGF21’s activity on angiogenesis, we performed H&E staining and IHC staining of CD34-positive vascular cells, and found that FGF21 increased the density of blood vessels in the flap tissue. We then investigated whether FGF21 modulates MMP9, VEGF, and Cadherin5, which are known to enhance angiogenesis^42,43^. MMP9 promotes the dissociation of cell junctions between mature vascular cells^44^, VEGF contributes to multiple processes of angiogenesis (particularly mitosis of vascular cells) and Cadherin5 promotes the formation and maturation of neovascularization^45^. Our results showed that with FGF21 treatment, MMP9, VEGF and Cadherin5 were all increased in the ischemic flap tissue. This suggests that FGF21 is a strong promoter of angiogenesis in the rat random flap model.

Next, we evaluated whether oxidative stress, a key determinant of tissue survival, is affected by FGF21. In the injury and repair process, when blood flow of ischemic tissue is resumed, oxygen molecules brought by the blood easily react to form superoxide anion which undergoes lipid peroxidation with the cell membrane and destroys it, causing cell-death tissue necrosis and producing MDA in the process^46,47^. Previous studies have shown that oxidative stress can be depressed by autophagy enhancement^48^. Thus we hypothesized that FGF21 may promote flap survival via inhibiting oxidative stress accumulation. Here, we showed via both IHC and WB that FGF21 increases the expression of SOD1, eNOS, and HO1, which are known to alleviate oxidative stress and reduced the level of MDA. Therefore, our results demonstrate that FGF21 is an inhibitor oxidative stress in random skin flaps.

Several studies have reported that FGF21 is also capable of inhibiting apoptosis^49,50^. For example, FGF21 depressed the level of apoptosis through activating the PI3K/Akt signaling pathway in a hypoxic-ischemic brain injury model of neonatal rats^51^. Apoptosis play a major role in the viability of ischemic skin flaps, so we hypothesized that FGF21 may have an inhibitory effect on apoptosis in our experimental model. From our TUNEL staining results, we found that flaps treated with FGF21 exhibited reduced DNA damage in cells, which may be a result of inhibited apoptosis. During apoptosis, various cellular stresses activate the mitochondria-mediated apoptotic pathway, leading to the release of CYC from mitochondria, which activates the caspase cascade and the final CASP3 as an executor of apoptosis^52^. Moreover, Bax is a pro-apoptotic protein that regulates the release of CYC^53^. Our molecular studies showed that FGF21 reduced the expression of CASP3, Bax, and CYC in ischemic flap tissues, suggesting that FGF21 inhibits apoptosis in rat random flaps.

Autophagy, a major process of degrading intracellular waste, is a known target of FGF21 and a known modulator of angiogenesis, apoptosis, and oxidative stress. It plays an essential role in maintaining intracellular homeostasis, and, in the setting of tissue damage, it alleviates the accumulation of toxins and waste released by damaged intracellular organelles^54^. In order to further study the mechanism of FGF21 promoting flap survival, the role of autophagy in the flap model was evaluated. Mechanistically, autophagy is responsible for degrading excess or severely damaged biomacromolecules and organelles by lysosome pathways via autophagosome formation, fusion of autophagosome and lysosome, and degradation of autophagic substrates^27^. In our present work, we showed that FGF21 leads to increased markers of autophagy activity. Moreover, after autophagy was inhibited with 3MA, angiogenesis in the flap tissue was reduced, oxidative stress and apoptotic activities were enhanced, and flap survival rate was decreased. Together, these results demonstrate that FGF21 enhanced angiogenesis, inhibited apoptosis and inhibited oxidative stress augmenting autophagy, ultimately improving skin flap survival.

To further elucidate the mechanism of action of FGF21 and how it promotes skin flap survival, we also explored upstream mechanisms of autophagy activity. Autophagy is thought to be regulated by post-transcriptional mechanisms, in which transcription factors play an essential role^55^. Recently, transcription factor EB (TFEB), a member of the MiT/TFE subfamily of the helix-loop-helix (bHLH) transcription factor family, has been shown to exert crucial effects in promoting autophagy-related transcriptional regulation^56,57^. Therefore, the relationship between tissue TFEB and autophagy activity was investigated in this study. Mechanistically, in the cytoplasm, phosphorylated TFEB and molecular chaperone 14-3-3 combine to form the TFEB-14-3-3 complex; when cells are stimulated by environmental signals (such as starvation, toxicity and oxidation), dephosphorylation of TFEB causes its dissociation from the TFEB-14-3-3 complex^58^. Then, free TFEB enters the nucleus and binds to many coordinated lysosomal expression and regulation (CLEAR) elements in DNA sequences which are related to autophagy, namely GTCACGTGAC. This initiates expression of autophagosome forming proteins LC3, Beclin1, and VPS34, and autophagy-related lysosomal functional proteins, such as CTSD^57^. In this study, FGF21 was shown to increase TFEB expression, and that autophagy activity in the flap model is regulated by TFEB. Taken together, our results show that FGF21 promotes autophagy via increasing the level of TFEB.

We also investigated how FGF21 regulates TFEB levels. Current studies have shown that intracellular TFEB is activated by various factors such as intracellular trophic disorders, pathogen invasion, macromolecular metabolic disorders and others^58^. These factors are related to nutritional deficiency caused by insufficient blood supply. In hypoxic, ischemic and hypoglycemic conditions, intracellular ATP reduction or increased AMP/ATP ratio can activate the phosphorylation of AMPK, which regulates cell metabolism through its downstream signaling pathways^59^. In the cytoplasm, the activation of the AMPK-mTOR pathway induces dephosphorylation of Ser211 of TFEB, leading to the dissociation of the TFEB-14-3-3 complex, allowing free TFEB to enter the nucleus^60^. In the nucleus, the activation of AMPK-FoxO3a-SPK2-CARM1 signaling cascade increases the level of CARM1, a co-activator of TFEB, which binds to TFEB and methylates promoter sequences, thereby inducing efficient transcription of intracellular autophagic genes^61^. In the present study, we demonstrated that both AMPK-mTOR and AMPK-FoxO3a-SPK2-CARM1 signaling pathways are activated after treating flaps with FGF21. Furthermore, compound C, an AMPK blocker, inhibited the FGF21-mediated activation of these signaling pathways. Together, our results confirmed that FGF21 activated TFEB in skin flaps through AMPK-FoxO3a-SPK2-CARM1 and AMPK-mTOR signaling pathways. Notably, the effect of FGF21 on AMPK-mTOR signaling is inconsistent across various studies. In neurons of Alzheimer’s disease, FGF21 was found to induce the activation in AMPK-mTOR signaling^62^, which is consistent with our findings. However, AMPK-mTOR signaling has also been shown to be inhibited by FGF21 in pancreatic islet cells^30,63^. The mechanisms of the differential effects of FGF21 in various cell types are unknown, which need further investigation. Of note, Chen et al. reported that FGF21 promoted autophagy in the liver via PP2A-mediated TFEB activation independent of AMPK. Whether FGF21 enhanced TFEB mediated autophagy through PP2A signaling in ischemic flaps should be explored in future studies^64^.

Naturally, there are several limitations of the present study that still need to be further investigated. For example, formation of double-membrane vesicles or autophagosomes is the key feature of macroautophagy, and electron microscope (EM) examination is a superior approach to identify these sub-cellular organelles compared with immunofluorescence staining of LC3II. Therefore, EM examination is necessary to be performed in the future. Moreover, in our study, a single dose of FGF21 was administered right after injury; the optimal dose and schedule of FGF21 should further evaluated to maximize translational and therapeutic value.

In conclusion, our studies showed that FGF21 activates AMPK-FoxO3a-SPK2-CARM1 and AMPK-mTOR signaling pathways, leading to increased intracellular TFEB expression, thereby augmenting tissue autophagy in ischemic random flaps. Higher levels of autophagy then enhance angiogenesis, inhibit apoptosis, and reduce oxidative stress, ultimately leading to increased flap viability. Together, these results provide strong evidence of FGF21’s therapeutic benefit for random-pattern skin flaps with novel mechanistic insight, highlighting FGF21’s potential for clinical translation pending further evaluation.

## Supplementary information

Supplementary Fig

Supplementary Figure Legend

cddis-author-contribution-form

## References

[1] McGregor IA, Morgan G (1973). Axial and random pattern flaps. Br. J. Plast. Surg..

[2] Kelly CP, Gupta A, Keskin M, Jackson IT (2010). A new design of a dorsal flap in the rat to study skin necrosis and its prevention. J. Plast., Reconstructive Aesthetic Surg..

[3] Russo CR, Leite MT, Gomes HC, Ferreira LM (2006). Transcutaneous electrical nerve stimulation in viability of a random skin flap in nicotine-treated rats. Ann. Plast. Surg..

[4] Wu H (2019). Trehalose promotes the survival of random-pattern skin flaps by TFEB mediated autophagy enhancement. Cell Death Dis..

[5] Li J (2019). Betulinic acid enhances the viability of random-pattern skin flaps by activating autophagy. Front. Pharmacol..

[6] Maulik N (1998). Ischemic preconditioning attenuates apoptotic cell death associated with ischemia/reperfusion. Mol. Cell Biochem..

[7] Basu G (2014). Prevention of distal flap necrosis in a rat random skin flap model by gene electro transfer delivering VEGF(165) plasmid. J. Gene Med..

[8] Kim TK (2009). The effects of botulinum toxin A on the survival of a random cutaneous flap. J. Plast., Reconstructive Aesthetic Surg..

[9] Xie XG, Zhang M, Dai YK, Ding MS, Meng SD (2015). Combination of vascular endothelial growth factor-loaded microspheres and hyperbaric oxygen on random skin flap survival in rats. Exp. Ther. Med..

[10] Fayazzadeh E (2016). Fibroblast growth factor-1 vs. fibroblast growth factor-2 in ischemic skin flap survival in a rat animal model. World J. Plast. Surg..

[11] Fujihara Y, Koyama H, Nishiyama N, Eguchi T, Takato T (2005). Gene transfer of bFGF to recipient bed improves survival of ischemic skin flap. Br. J. Plast. Surg..

[12] Yaqoob U (2014). FGF21 promotes endothelial cell angiogenesis through a dynamin-2 and Rab5 dependent pathway. PLoS ONE.

[13] Nishimura T, Nakatake Y, Konishi M, Itoh N (2000). Identification of a novel FGF, FGF-21, preferentially expressed in the liver 1. BBA - Gene Struct. Expr..

[14] Seo JA, Kim NH (2012). Fibroblast growth factor 21: a novel metabolic regulator. Diabetes Metab. J..

[15] Alexei K (2007). The metabolic state of diabetic monkeys is regulated by fibroblast growth factor-21. Endocrinology.

[16] So WY, Leung PS (2016). Fibroblast growth factor 21 as an emerging therapeutic target for type 2 diabetes mellitus. Medicinal Res. Rev..

[17] So WY, Cheng Q, Xu A, Lam KS, Leung PS (2015). Loss of fibroblast growth factor 21 action induces insulin resistance, pancreatic islet hyperplasia and dysfunction in mice. Cell Death Dis..

[18] Xu P (2016). Fibroblast growth factor 21 attenuates hepatic fibrogenesis through TGF-beta/smad2/3 and NF-kappaB signaling pathways. Toxicol. Appl. Pharmacol..

[19] Wente W (2006). Fibroblast growth factor-21 improves pancreatic beta-cell function and survival by activation of extracellular signal-regulated kinase 1/2 and Akt signaling pathways. Diabetes.

[20] Yan X (2018). Fibroblast growth factor 21 inhibits atherosclerosis in apoE-/- mice by ameliorating Fas-mediated apoptosis. Lipids Health Dis..

[21] Huang W (2019). Fibroblast growth factor 21 enhances angiogenesis and wound healing of human brain microvascular endothelial cells by activating PPARgamma. J. Pharmacol. Sci..

[22] Zhang Xiang, Yang Luo, Xu Xiongfeng, Tang Fengjuan, Yi Peng, Qiu Bo, Hao Yarong (2019). A review of fibroblast growth factor 21 in diabetic cardiomyopathy. Heart Failure Reviews.

[23] Prado RP, Liebano RE, Hochman B, Pinfildi CE, Ferreira LM (2006). Experimental model for low level laser therapy on ischemic random skin flap in rats. Acta Cirurgica Brasileira.

[24] Kaminski KA, Bonda TA, Korecki J, Musial WJ (2002). Oxidative stress and neutrophil activation—the two keystones of ischemia/reperfusion injury. Int. J. Cardiol..

[25] Gottlieb RA, Burleson KO, Kloner RA, Babior BM, Engler RL (1994). Reperfusion injury induces apoptosis in rabbit cardiomyocytes. J. Clin. Investig..

[26] Zhai M (2017). Melatonin ameliorates myocardial ischemia reperfusion injury through SIRT 3‐dependent regulation of oxidative stress and apoptosis. J. Pineal Res..

[27] Parzych KR, Klionsky DJ (2013). An overview of autophagy: morphology, mechanism, and regulation. Antioxid. Redox Signal.

[28] Zhu S (2016). FGF21 ameliorates nonalcoholic fatty liver disease by inducing autophagy. Mol. Cell Biochem.

[29] Rupérez C (2018). Autophagic control of cardiac steatosis through FGF21 in obesity-associated cardiomyopathy. Int. J. Cardiol..

[30] Leung PS (2019). FGF21 activation-mediated islet autophagy in Type 2 diabetes with pharmacotherapeutic potential. Future medicinal Chem..

[31] Ren Z (2019). Fibroblast growth factor-21 alleviates hypoxia/reoxygenation injury in H9c2 cardiomyocytes by promoting autophagic flux. Int. J. Mol. Med..

[32] Lin J (2019). Therapeutic potential of pravastatin for random skin flaps necrosis: involvement of promoting angiogenesis and inhibiting apoptosis and oxidative stress. Drug Des., Dev. Ther..

[33] Lin J (2018). Salvianolic acid B promotes the survival of random-pattern skin flaps in rats by inducing autophagy. Front. Pharmacol..

[34] Abraham A (2016). Laser Doppler flare imaging and quantitative thermal thresholds testing performance in small and mixed fiber neuropathies. PLoS ONE.

[35] Seyed Jafari SM (2017). Improvement of flap necrosis in a rat random skin flap model by in vivo electroporation-mediated HGF gene transfer. Plast. Reconstructive Surg..

[36] Fukunaga Y (2017). Topical application of nitrosonifedipine, a novel radical scavenger, ameliorates ischemic skin flap necrosis in a mouse model. Wound Repair Regeneration.

[37] Zhou KL, Zhang YH, Lin DS, Tao XY, Xu HZ (2016). Effects of calcitriol on random skin flap survival in rats. Sci. Rep..

[38] Kannan K, Jain SK (2000). Oxidative stress and apoptosis. Pathophysiology.

[39] Gottlieb RA, Engler RL (1999). Apoptosis in myocardial ischemia-reperfusion. Ann. New Y. Acad. Sci..

[40] Fisher FM, Maratos-Flier E (2016). Understanding the physiology of FGF21. Annu Rev. Physiol..

[41] Yu Y (2015). Fibroblast growth factor (FGF21) protects mouse liver against D-galactose-induced oxidative stress and apoptosis via activating Nrf2 and PI3K/Akt pathways. Mol. Cell Biochem..

[42] Folkman J (2006). Angiogenesis. Annu Rev. Med..

[43] Folkman J (2007). Angiogenesis: an organizing principle for drug discovery?. Nat. Rev. Drug Discov..

[44] Li X (2018). Ten-eleven translocation 2 demethylates the MMP9 promoter, and its down-regulation in preeclampsia impairs trophoblast migration and invasion. J. Biol. Chem..

[45] Carmeliet P, Collen D (2000). Molecular basis of angiogenesis. Role of VEGF and VE-cadherin. Ann. New Y. Acad. Sci..

[46] Halliwell B, Chirico S (1993). Lipid peroxidation: its mechanism, measurement, and significance. Am. J. Clin. Nutr..

[47] Tsikas D (2017). Assessment of lipid peroxidation by measuring malondialdehyde (MDA) and relatives in biological samples: analytical and biological challenges. Anal. Biochem..

[48] Filomeni G, De Zio D, Cecconi F (2015). Oxidative stress and autophagy: the clash between damage and metabolic needs. Cell death Differ..

[49] Tabari FS (2019). The roles of FGF21 in atherosclerosis pathogenesis. Rev. Endocr. Metab. Disord..

[50] Wang HW (2019). FGF21 protects against hypoxia injury through inducing HSP72 in cerebral microvascular endothelial cells. Front. Pharmacol..

[51] Ye L (2019). FGF21 promotes functional recovery after hypoxic-ischemic brain injury in neonatal rats by activating the PI3K/Akt signaling pathway via FGFR1/beta-klotho. Exp. Neurol..

[52] Elmore S (2007). Apoptosis: a review of programmed cell death. Toxicol. Pathol..

[53] Pietenpol JA, Stewart ZA (2002). Cell cycle checkpoint signaling: cell cycle arrest versus apoptosis. Toxicology.

[54] Levine B, Klionsky DJ (2004). Development by self-digestion: molecular mechanisms and biological functions of autophagy. Dev. Cell.

[55] Feng Y, Yao Z, Klionsky DJ (2015). How to control self-digestion: transcriptional, post-transcriptional, and post-translational regulation of autophagy. Trends Cell Biol..

[56] Settembre C (2011). TFEB links autophagy to lysosomal biogenesis. Science.

[57] Medina DL (2015). Lysosomal calcium signalling regulates autophagy through calcineurin and TFEB. Nat. Cell Biol..

[58] Raben N, Puertollano R (2016). TFEB and TFE3: linking lysosomes to cellular adaptation to stress. Annu. Rev. Cell Developmental Biol..

[59] Herzig S, Shaw RJ (2018). AMPK: guardian of metabolism and mitochondrial homeostasis. Nat. Rev. Mol. Cell Biol..

[60] Young NP (2016). AMPK governs lineage specification through Tfeb-dependent regulation of lysosomes. Genes Dev..

[61] HJ S (2016). AMPK-SKP2-CARM1 signalling cascade in transcriptional regulation of autophagy. Nature.

[62] Kuhla A (2019). Metformin therapy aggravates neurodegenerative processes in ApoE-/- mice. J. Alzheimer’s. Dis..

[63] Cheng, S. T. W., Li, S. Y. T. & Leung, P. S. Fibroblast growth factor 21 stimulates pancreatic islet autophagy via inhibition of AMPK-mTOR signaling. *Int. J. Mol. Sci.* 2019, **20** 2517 (2019). 10.3390/ijms20102517 PMC6567208 31121855

[64] Chen L (2017). Fasting-induced hormonal regulation of lysosomal function. Cell Res..

