# Supplementary figures and images for: FGF21 augments autophagy in random-pattern skin flaps via AMPK signaling pathways and improves tissue survival
Source: Cell Death Dis. 2019 Nov 18;10(12):872. doi: 10.1038/s41419-019-2105-0 (PMC6861244; doi:10.1038/s41419-019-2105-0)

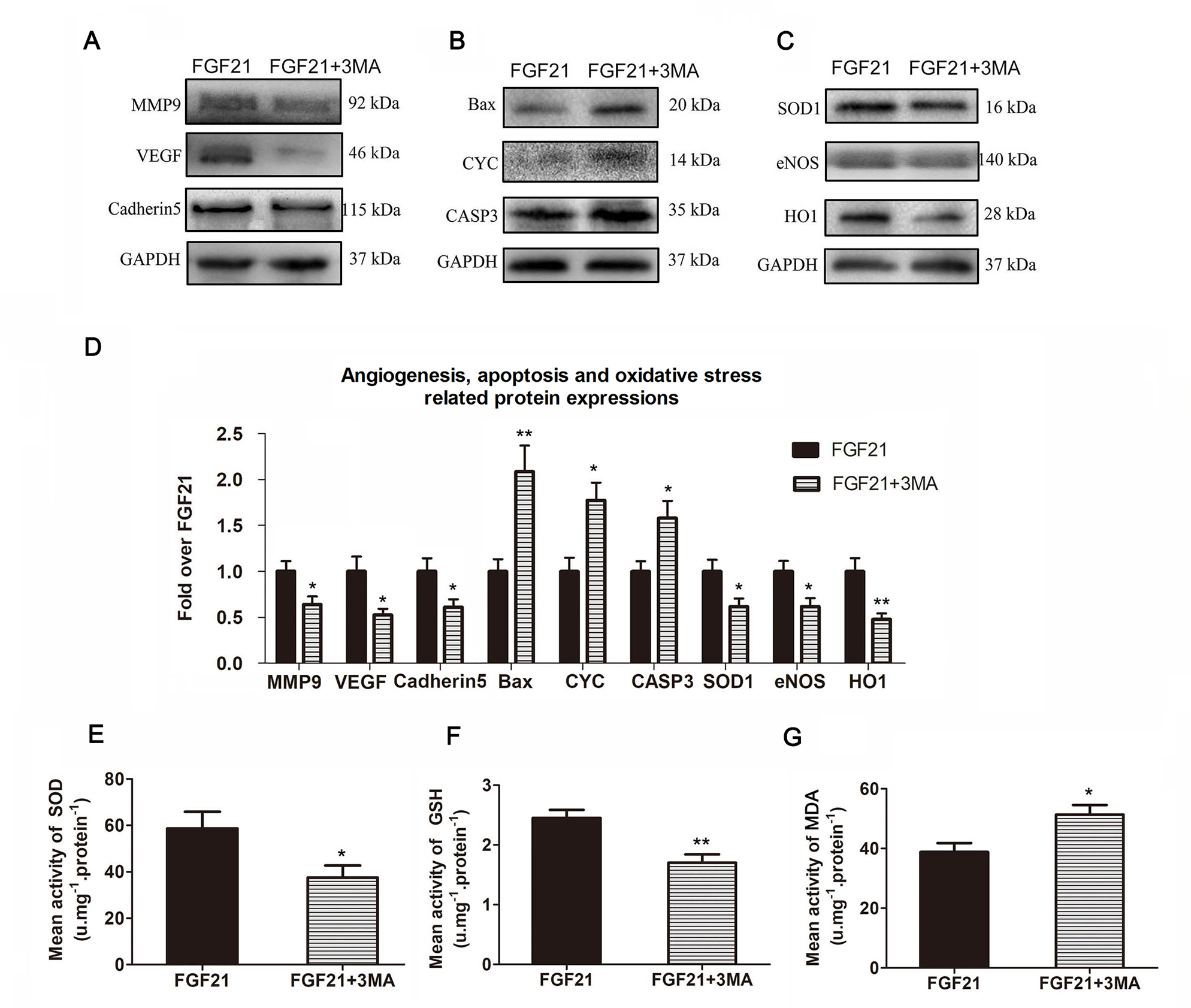

Supplement: Supplementary file 1 — Supplementary Fig [file 41419_2019_2105_MOESM1_ESM.png]
