# Supplementary material for: FGF21 augments autophagy in random-pattern skin flaps via AMPK signaling pathways and improves tissue survival
Source: Cell Death Dis. 2019 Nov 18;10(12):872. doi: 10.1038/s41419-019-2105-0 (PMC6861244; doi:10.1038/s41419-019-2105-0)
Supplement: Supplementary file 2 — Supplementary Figure Legend [file 41419_2019_2105_MOESM2_ESM.doc]

**Supplementary figure.** 3MA reversed effects of FGF21 on angiogenesis, oxidative stress and apoptosis in flaps. On the 7^th^ day after operation of random flap, the rats were sacrificed in the FGF21 group and FGF21+3MA group, and the samples were harvested for assessment. (**A, B, C**) Western blotting for levels of angiogenic proteins (MMP9, VEGF and Cadherin 5), apoptotic proteins (Bax, CYC and CASP3) and oxidative stress proteins (SOD1, eNOS and HO1), which was corrected by GAPDH as internal control. (**D**) Histogram showing the quantification of angiogenic proteins (MMP9, VEGF and Cadherin 5), apoptotic proteins (Bax, CYC and CASP3) and oxidative stress proteins (SOD1, eNOS and HO1) detected by Western blotting. (**E**) Evaluation of SOD activity by the assay of the xanthine oxidase method. (**F**) Assessment of GSH activity using modified 5,5’-dithiobis method. (**G**) Measurement of MDA content by the modified TBA test. Significance: **p* < 0.05 and ** *p* < 0.01 vs the FGF21 group. Data were expressed as means ± SEM (n = 6 per group).
