# Supplementary material for: FGF21 augments autophagy in random-pattern skin flaps via AMPK signaling pathways and improves tissue survival
Source: Cell Death Dis. 2019 Nov 18;10(12):872. doi: 10.1038/s41419-019-2105-0 (PMC6861244; doi:10.1038/s41419-019-2105-0)
Supplement: Supplementary file 3 — cddis-author-contribution-form [file 41419_2019_2105_MOESM3_ESM.pdf]

DECLARATION OF CONTRIBUTIONS TO ARTICLE

ADMC

Manuscript Number: CDDIS-19-2699R

Journal Name: *Cell Death & Disease* (the 'Journal')

Proposed Title of the Contribution:

FGF21 Augments Autophagy in Random-Pattern Skin Flaps via AMPK Signaling Pathways and Improves Tissue Survival (the 'Contribution)

Author(s):

Kailiang Zhou,Huanwen Chen,Jinti Lin, Hui Xu, Hongqiang Wu, Guodong Bao, Jiangfeng Li, Xiangyang Deng, Xiaolong Shui, Weiyang Gao, Jian Ding, Jian Xiao, Huazi Xu (the 'Authors')

For all *CDDis* articles, each person named as an author in the published version must be able to show he or she has contributed substantially to the article.

Authorship credit should be based on 1) substantial contributions to conception and design, acquisition of data, or analysis and interpretation of data; 2) drafting the article or revising it critically for important intellectual content; and 3) final approval of the version to be published. Authors should meet conditions 1, 2 and 3.

Any person who cannot be shown to have made a substantial contribution to the article cannot be listed as an author in the final version. The name of any person who is deemed to have made a minor contribution can, however, appear in the Acknowledgments section of the article.

Please complete the table below to indicate the contributions of all named authors to the manuscript.

Author Full Name:	Specification of Contribution to the Manuscript:
Kailiang Zhou	wrote the manuscript text；prepared figures and collected samples；designed the experiment；revised manuscript；reviewed and approved the final manuscript
Huanwen Chen	wrote the manuscript text；revised manuscript；reviewed and approved the final manuscript
Jinti Lin	wrote the manuscript text；reviewed and approved the final manuscript
Hui Xu	prepared figures and collected samples；reviewed and approved the final manuscript
Hongqiang Wu	prepared figures and collected samples；reviewed and approved the final manuscript
Guodong Bao	prepared figures and collected samples；reviewed and approved the final manuscript
Jiangfeng Li	prepared figures and collected samples；reviewed and approved the final manuscript
Xiangyang Deng	analyzed data；reviewed and approved the final manuscript
Xiaolong Shui	analyzed data；reviewed and approved the final manuscript
Weiyang Gao	revised manuscript；reviewed and approved the final manuscript
Jian Ding	designed the experiment；revised manuscript；reviewed and approved the final manuscript
Jian Xiao	designed the experiment；revised manuscript；reviewed and approved the final manuscript
Huazi Xu	designed the experiment；revised manuscript；reviewed and approved the final manuscript

Please complete the table below to indicate the contributions of all named authors to the figures.

Figure 1:

Kailiang Zhou, Hui Xu, Hongqiang Wu, Guodong Bao, Jiangfeng Li collected samples and prepared the figure. Xiangyang Deng and Xiaolong Shui analyzed data.

Figure 2:

Kailiang Zhou, Hui Xu, Hongqiang Wu, Guodong Bao, Jiangfeng Li collected samples and prepared the figure. Xiangyang Deng and Xiaolong Shui analyzed data.

Figure 3:

Kailiang Zhou, Hui Xu, Hongqiang Wu, Guodong Bao, Jiangfeng Li collected samples and prepared the figure. Xiangyang Deng and Xiaolong Shui analyzed data.

Figure 4:

Kailiang Zhou, Hui Xu, Hongqiang Wu, Guodong Bao, Jiangfeng Li collected samples and prepared the figure. Xiangyang Deng and Xiaolong Shui analyzed data.

Figure 5:

Kailiang Zhou, Hui Xu, Hongqiang Wu, Guodong Bao, Jiangfeng Li collected samples and prepared the figure. Xiangyang Deng and Xiaolong Shui analyzed data.

Figure 6:

Kailiang Zhou, Hui Xu, Hongqiang Wu, Guodong Bao, Jiangfeng Li collected samples and prepared the figure. Xiangyang Deng and Xiaolong Shui analyzed data.

Signed for and on behalf of the Author(s):	Print Name:	Date:
Huazi Xu	Huazi Xu	2019-10-05
